# Elites are people, too: The effects of threat sensitivity on policymakers’ spending priorities

**DOI:** 10.1371/journal.pone.0193781

**Published:** 2018-04-10

**Authors:** Kevin Arceneaux, Johanna Dunaway, Stuart Soroka

**Affiliations:** 1 Department of Political Science, Temple University, Philadelphia, Pennsylvania, United States of America; 2 Department of Communication, Texas A&M University, College Station, Texas, United States of America; 3 Department of Communication Studies, University of Michigan, Ann Arbor, Michigan, United States of America; Rice University, UNITED STATES

## Abstract

Recent research suggests that psychological needs can influence the political attitudes of ordinary citizens, often outside of their conscious awareness. In this paper, we investigate whether psychological needs also shape the spending priorities of political elites in the US. Most models of policymaking assume that political elites respond to information in relatively homogeneous ways. We suggest otherwise, and explore one source of difference in information processing, namely, threat sensitivity, which previous research links to increased support for conservative policy attitudes. Drawing on a sample of state-level policymakers, we measure their spending priorities using a survey and their level of threat sensitivity using a standard psychophysiological measure (skin conductance). We find that, like ordinary citizens, threat sensitivity leads even state-level policymakers to prioritize spending on government polices that are designed to minimize threats.

## Introduction

Democracies are supposed to produce policies that align with the public’s preferences. Although the ideal form of democracy provides ordinary citizens direct control over policy and governance, in most modern democracies policy making is left to politicians and their staffs. In recognition of the public’s putative centrality in democratic governance, studies of policymaking treat politicians and their staff as well-honed, career-minded machines who strategically select behavioral responses to electorally created incentives [1–6]. Nonetheless, politicians (and their staff) do not always have an accurate perception of what their constituents want [7, 8] and fill in the gaps by projecting their preferences onto their constitutes [9, 10]. As a result, policymaking can end up reflecting politicians’ wants and desires, even if elected officials are trying to be faithful representatives to their constituents.

It is, therefore, crucial to understand how politicians form policy preferences. Canonical models of policymaking presume that policymakers process information in a uniform fashion—given the same facts, they should reach the same conclusions. Differences in preferences, then, should simply reflect differences in political philosophies. However, recent research shows that psychological biases are more important for elite decision making than canonical models of policymaking presume [11–13]. Although there is an understandable tendency to treat policymakers as special—after all, they are an elite group of policymakers—they are also only human. Rather than treating policymakers as well-honed machines who process information in a uniform way, we look to the study of how ordinary citizens form political preferences to gain insight into the psychology of policymaking.

According to decades of research, ordinary people do not process information uniformly. Differences in genetics coupled with early life experiences lead to differences in the architecture of brain and, thus, the way in which people process external information [14–16]. In the realm of politics, deep-seated differences in how individuals process information profoundly shapes political attitudes [17]. A burgeoning area of research focuses particular attention to how people process perceived threats. Some people greet reports of terrorism, crime, and the like with a great deal of alarm, while others take them in stride. Recent work suggests that people who are highly sensitive to threats come to see the world as a dangerous and threatening place [18] and tend to see conservative policies—such as adherence to conventional morality and support for a strong military—as a way to cope with and manage the dangerous and threatening world in which they live [19–21]. These tendencies are evident in the association between conservatism and higher levels of electrodermal activity (i.e., sweating) in response to threatening images [22–24]. Studies of neural activity suggest that the brains of people who hold conservative attitudes may be more attuned than liberals’ brains to potentially threatening stimuli [25, 26].

## Data and results

This research was approved by Texas A&M IRB and was administered by Professor Johanna Dunaway who is on faculty at Texas A&M.

**Fig 1:**
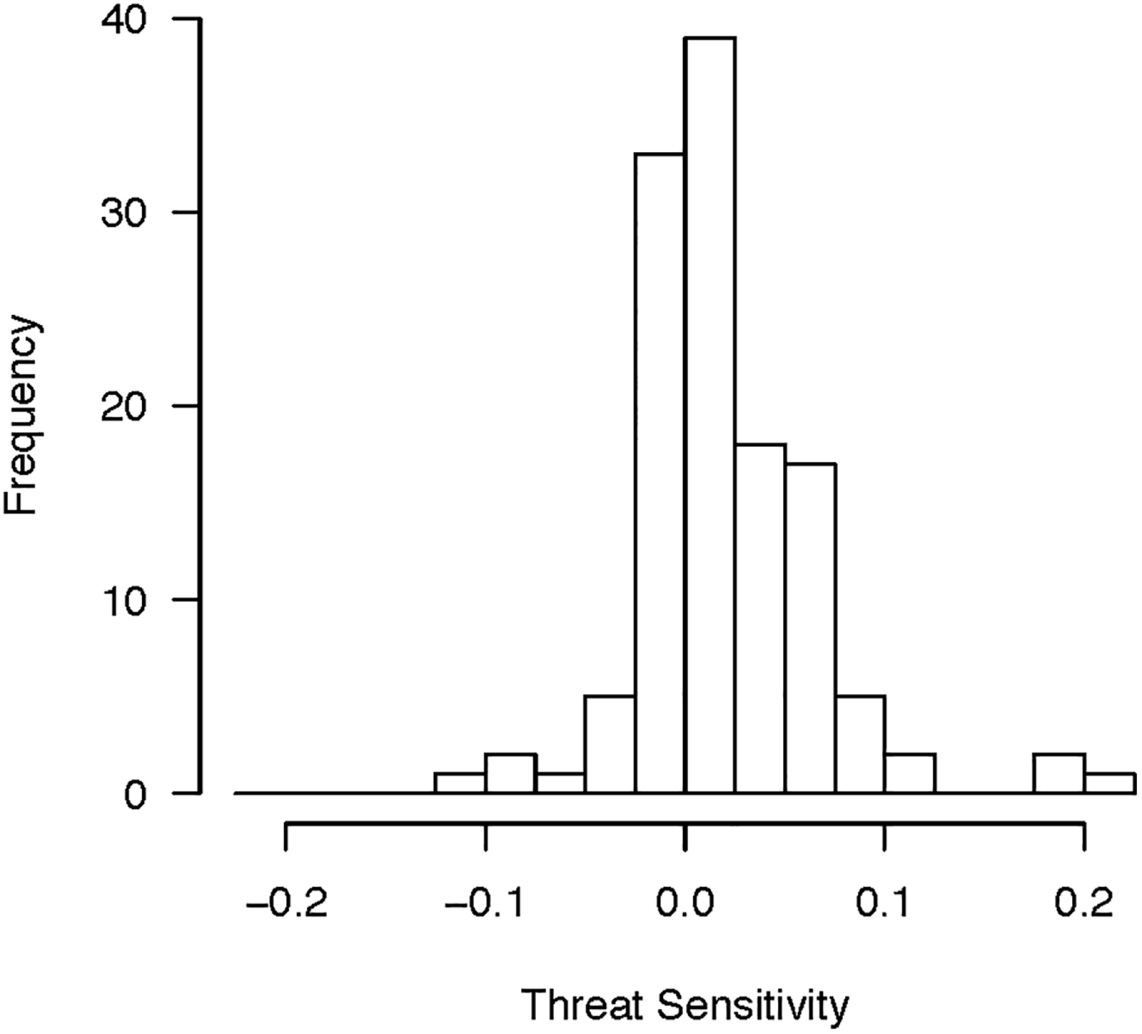
The distribution of threat sensitivity.

We extend this line of research to the study of policymakers in the United States. We are aware of no study that investigates this central claim. Although one study shows that center-right political elites in Iceland offered more conservative policy opinions when induced to feel threat [27], it leaves unresolved whether politicians differ in how they process and respond to threatening stimuli. We evaluate the effects of threat sensitivity on policymakers’ policy preferences by recruiting 173 state legislators and their staff at the 2016 National Conference of State Legislators to participate in a brief study. The study consisted of a survey that measured state policymakers’ spending preferences and a standard protocol for measuring physiological responses to threatening stimuli [23]. In particular, we measured threat sensitivity by sitting participants before a laptop, behind a privacy partition, and asked them to wear noise-canceling headphones to minimize distractions. Biosensors were attached to the first to third fingers on their non-dominant hand to capture skin conductance. Participants were then presented with four images in succession. The first two images were selected to be neutral and non-affective so that we could capture participants’ baseline physiological activity (a basket and a spoon, presented in random order). The second set of images were selected for their threatening content (a snake lunging at the camera and a close-up of a angry, barking guard dog, presented in random order). The skin conductance levels (SCL) captured during the images allow us to measure participants’ electrodermal activity when faced with threatening non-political images. Because electrodermal activity is difficult to regulate consciously, it offers a valid and reliable measure of people’s sympathetic nervous system response [28]. See S1 File for details about the survey and threat sensitivity protocol. Fig 1 shows the distribution of our measure capturing threat sensitivity, which is the difference in SCL observed during the two threatening photos and the SCL observed during the two neutral photos. Higher values indicate greater sensitivity to threat. The distribution clearly leans to the right (i.e., towards threat sensitivity), but there is a good bit of variation as well.

The survey asked policymakers to indicate what percent of their state budget they would devote to six policy domains that consistently divide liberals and conservatives: assistance to the poor, elementary and secondary education, counter-terrorism, health care, higher education, and police and public safety. Four of these tend to be liberal priorities: spending on education, higher education, health and the poor; two others tend to be conservative priorities: spending on defense and terror. Our measure of spending preferences captures the conservative-liberal difference by asking policymakers to indicate the percent of the state budget they would allocate to each domain; and then capturing policymakers’ spending priorities by taking the average spending preference on the two conservative priorities and subtracting from it the average spending preference on the four liberal priorities. We find suggestive evidence that threat sensitivity correlates with a preference for devoting a higher portion of the state budget to conservative spending priorities vis-a-vis liberal ones (*β* = 78.1, *SE* = 133.9, *P* = 0.28), even after controlling for participants’ partisan affiliation (*β* = 77, *SE* = 133.2, *P* = 0.29). (See Table B in S1 File for details). Although these findings corroborate findings from layperson samples that threat sensitivity correlates with conservative policy preferences [20], we cannot rule out sampling variability as a possible explanation.

Although the six policies that are part of this general measure clearly relate to conservative-liberal policy priorities, we expect that some are at best only loosely related to threat sensitivity. Pushing beyond these preliminary but suggestive findings, then, we focused the analysis on the degree to which policymakers prioritize spending on counterterrorism to spending on assistance to the poor. We do so for three reasons. First, these policy domains are primarily national, not state-level, and, therefore, do not differ widely across states. Second, these issues lie at the center of partisan polarization in the US that previous scholars have traced to differences in threat sensitivity [29], and recent survey research shows that counterterrorism and welfare stand out in this regard [30]. Third, terrorism is the policy domain that should be most clearly rooted in concerns about a looming, external threat [31, 32]. Welfare, in contrast, is the social domain that is most clearly involves an empathetic giving to others, even in the face of moral risk. Consequently, the terror-welfare tradeoff—capturing spending on threat versus spending on targeted income redistribution—should be more directly connected to threat sensitivity than the general spending priorities measure.

**Fig 2:**
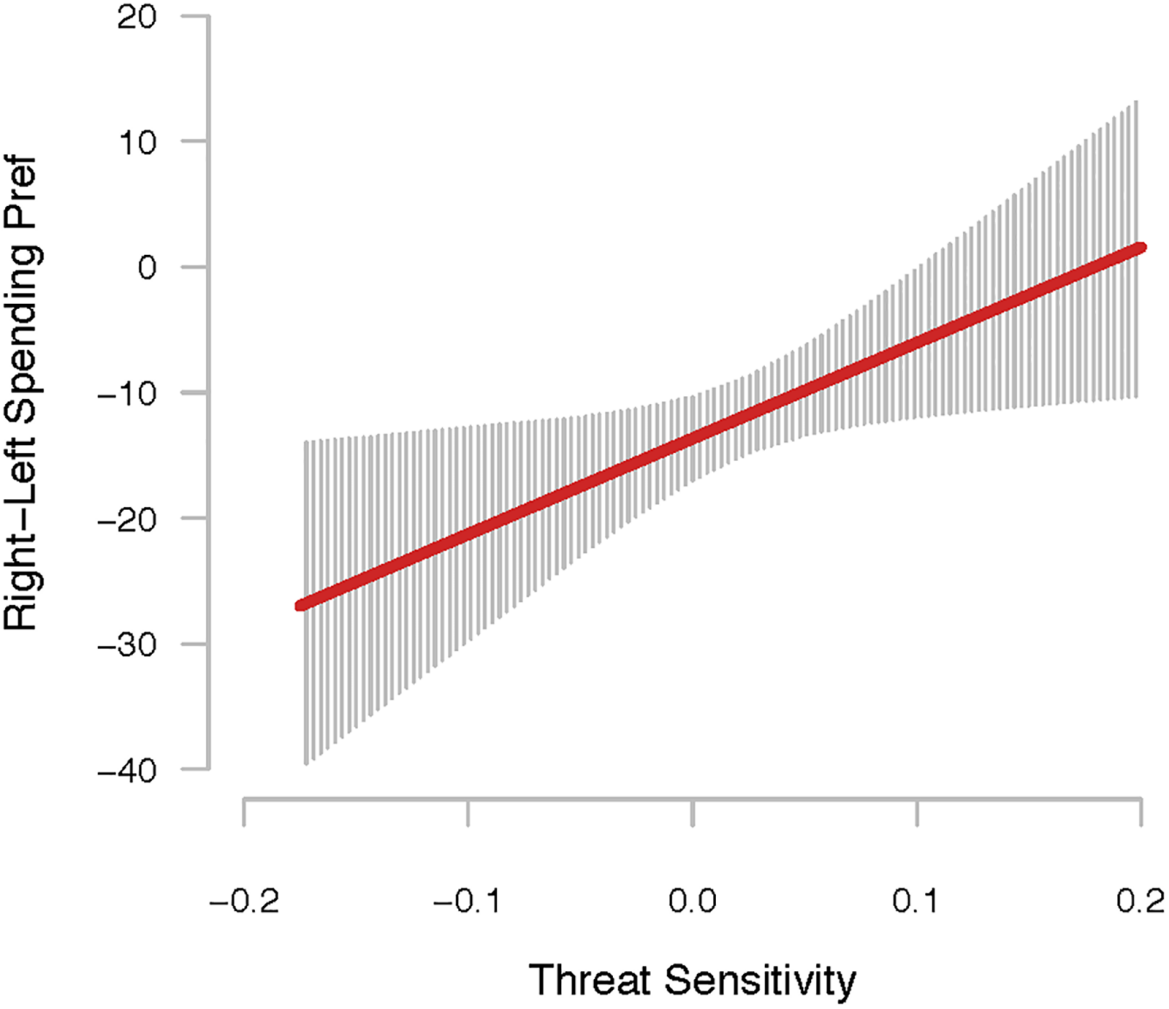
The relationship between threat sensitivity and right-left spending preferences.

The results reported in Table 1 confirm these expectations. Here we observe a positive and statistically significant relationship between threat sensitivity and policymakers’ tendency to prioritize counter-terrorism over welfare spending. This relationship is unaffected when partisan affiliation is added to the model (Column 2 of Table 1). Fig 2 plots the estimated relationship between threat sensitivity and counter-terrorism spending priority, from Model 2 of Table 1. Moving across the observed range of threat sensitivity is associated with a shift in a preference for prioritizing counter-terrorism over welfare spending by 30 percentage points. On a measure that ranges from -56 to +33, with a standard deviation of 16.2, this is a rather striking relationship.

**Table 1:** Terror-poor spending preferences and threat sensitivity.

	*Dependent variable*:	
	Spending on Terror—Poor Issues	
	(1)	(2)
Threat Sensitivity	76.865*	76.151*
	(33.519)	(32.386)
Democrat		−8.696*
		(3.846)
Republican		1.684
		(4.925)
Constant	−13.676***	−8.672*
	(1.741)	(3.387)
Observations	102	102
R^2^	0.050	0.131
Adjusted R^2^	0.040	0.105
Residual Std. Error	16.040 (df = 100)	15.494 (df = 98)
F Statistic	5.259* (df = 1; 100)	4.935** (df = 3; 98)

Note:

* p<0.05;

** p<0.01;

*** p<0.001

We regard the tradeoff between counterterrorism and welfare as the most relevant for our purposes, but note that our results are not dependent on these two domains alone. One obvious change is to replace counterterrorism with crime prevention. There will more regional variation in crime-related spending; and crime prevention is less directly connected to threat sensitivity in the existing literature. Even so, results using this slightly revised measure produce roughly similar results. We include those results in Table C in S1 File.

The empirical evidence that we present suggests that the genesis of policy elites’ spending preferences is more complicated than theoretical models assume. When these individuals are making budgetary tradeoffs, threat sensitivity correlates with prioritizing spending on counter-terrorism to welfare spending—the classic guns versus butter tradeoff.

These findings challenge the notion that elites’ preferences can be simply reduced to strategically selecting behavioral responses to environmentally created incentives as well as the notion that strong incentives for policy elites to be cold and calculating obviates the influence of psychological needs. Instead we find that, like ordinary citizens, threat sensitivity plays an important role in state-level policymakers decisions to prioritize spending on government polices that are designed to minimize threats. To be clear, this is not to say that these elites are not being “rational.” They could certainly be behaving rationally in the sense that they are connecting their psychological needs in a logical way to their policy preferences [21]. What these findings show is that elites—like all humans—vary in the way in which they evaluate the same information, leading them to adopt different policy preferences. Individual differences in information processing have not been incorporated into standard theoretical models of elite decision-making, and our findings suggest that perhaps they should. Doing so may provide insights into why some policymakers systematically perceive their constituents to be more conservative than they actually are [33].

Like all studies, ours has several limitations. First, in order to preserve anonymity, we did not collect personal information about these participants, such as the districts that they represent. It could be that particular constituencies elect representatives with psychological needs that mirror the modal member of their district. If so, it would support an “electoral connection” between constituents and representatives [4], albeit one that does not require the representative to behave in a strategic way. Second, we do not know the level of professionalism of the legislature in which these policymakers inhabit or their level of progressive ambition. Some state legislatures are highly professionalized miniature versions of the US Congress, while others are filled with mostly part-time citizen legislators [34]. Policymakers from less professionalized legislatures may behave in less strategic ways. On this point, individual legislators—irrespective of the type of legislature in which they work—vary in the degree to which they have ambitions to progress up the electoral ladder to a higher office [6]. Perhaps more ambitious individuals will behave in a more calculating fashion.

Furthermore, more research is needed regarding the psychophysiological measurement of threat sensitivity and, more generally, negativity bias in this domain of political science. We chose images that would directly tap into threat and, therefore, feelings of anxiety and fear. However, other researchers have used a variety of images to tap threat sensitivity that may also tap into disgust [23]—making those measures better thought of as a general indicator of negativity bias that includes threat and disgust sensitivity [20]. We find it noteworthy that our more tailored images point to the same conclusions as a more general measure of negativity bias. Nonetheless, we cannot speak to the specific effects of threat and disgust—whether both forms of negativity bias contribute equally to inducing conservative attitudes or whether they operate differently in different domains.

Despite these limitations, we believe that our study makes an important contribution to the study of elite behavior. Scholars should take the psychological needs of policymakers more seriously. In short, we find that policymakers are people, too. Additional research should be directed at understanding the conditions that moderate and mediate the influence of psychological needs on elite decision making and behavior.

## Supporting information

S1 File (PDF)

S1 Data (ZIP)
