# Supplementary material for: Elites are people, too: The effects of threat sensitivity on policymakers’ spending priorities
Source: PLoS One. 2018 Apr 10;13(4):e0193781. doi: 10.1371/journal.pone.0193781 (PMC5892868; doi:10.1371/journal.pone.0193781)
Supplement: S1 File — (PDF) [file pone.0193781.s001.pdf]

Supporting information (SI) for *Elites are People, Too*

Kevin Arceneaux[1*], Johanna Dunaway[2], Stuart Soroka[3]
1 Department of Political Science, Temple University,

2 Department of Communication, Texas A&M University,

3 Department of Communication Studies, University of Michigan,

*Corresponding author

E-Mail: `arceneau@temple.edu`

Recruitment and setting

Participants were voluntarily recruited from the attendees at the 2016 National Conference of State Legislators, held August 9-11 at the McCormick Convention Center in Chicago, IL. More than 2,000 elected officials and staff members annually attend this type of conference. Partnering with a small group of researchers from four other universities (Washington University in St. Louis, Northwestern University, University of California San Diego, and University of California Santa Barbara), and Laboratories of Democracy (http://www.labsofdemocracy.org/), we purchased a booth in the exhibit hall of the conference. We equipped the booth with four tables at which participants completed the survey portion on tablets, and a computer terminal with a privacy screen at which participants completed the physiological portion of the study (See Fig. 3).

Most of the research team was physically present in the booth at all times, rotating individual breaks for members as needed. Various team members alternated roles in the booth: recruiting, obtaining consent, assisting survey takers, and manning the physiological portion of the study. Our recruitment strategy took advantage of conference attendee foot traffic through the convention hall, inviting state legislators and their staffers to take our short survey as they passed the strategically located corner booth. Recruits were told their participation would help us with our research and that for participating they earn a free t-shirt (for the first 250 participants). We recruited 173 participants for this study, of which 102 completed all questions and psychophysiological task. Recruited participants were conference attendees (state legislators and staff members), at least 18 years of age.

Survey

After recruitment, members of the research team accompanied participants to tables in the booth to obtain informed consent for portions 1 and 2 of the study. Once subjects indicated their consent they were provided with tablets on which our survey was pre-loaded using the Qualtrics survey software application for mobile devices (http://www.qualtrics.com/research-suite/).

During Part 1, participants answered a series of survey questions about their basic demographics and their role/position in state government, views of social conservativism (using a battery of

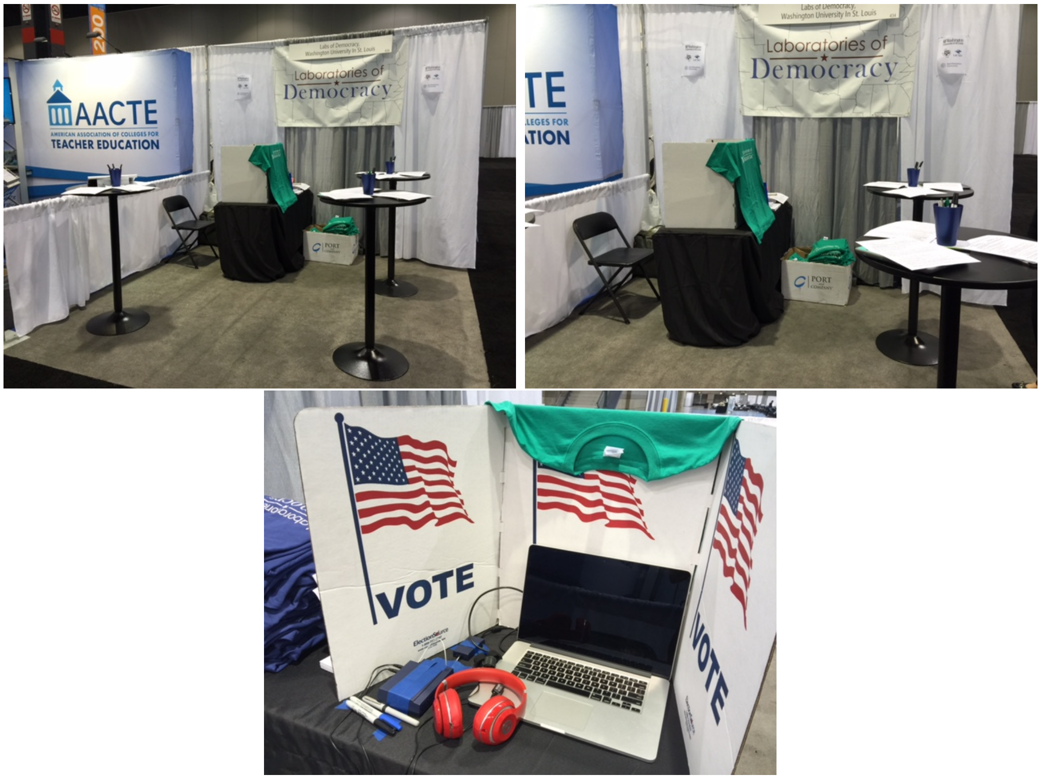

Figure A: Research booth at 2016 National Conference of State Legislators

questions about how to organize society), their spending priorities, and what types of news stories are relevant to their responsibilities and district.

After completing the survey, participants had the option to continue with Part 2 of the study, which involved moving to a computer in back of the booth (using a unique non-identifying code was used to match responses with the previous survey), where they view a series of images drawn from the International Affective Picture System (IAPS) database.

Survey questions used in the preceding analyses are as follows.

Basic demographics and partisanship

What is your gender? (Female / Male)

For which state do you work? (Drop down menu)

Which best describes your position? (State legislator / Legislative staff / Government or public employee / Other)

What party do you identify with? (Republican / Democratic / Independent or unaffiliated / Other)

Which chamber do you work for in your state? (Upper chamber / Lower chamber / Not applicable)

Spending priorities

Below are some broad policy areas. If it were up to you, what percent of the budget would you devote to each area? (answers recorded with a slider that went from 0 to 100): Police and Public Safety / Counter Terrorism / Elementary and Secondary Education / Higher Education / Highway Infrastructure / Health Care / Assistance for the Poor

Physiological measures

We base the physiological portion of our study on photos from the International Affective Picture System (IAPS). The IAPS is a large battery of still photos coded on several dimensions by expert coders. It is distributed by the Center for the Study of Emotion and Attention at the University of Florida (http://csea.phhp.ufl.edu/index.html), and used in a wide range of psychological and psychophysiological experiments.

The goal of the experiment is to examine threat sensitivity using individuals' reactions to an array of still photos. To capture physiological responses to IAPS photos we invited participants to take place in an optional, second-phase of the study immediately following the survey portion. Participants viewed a short battery of four still photos, on a computer monitor behind a privacy partition, and wearing noise-canceling headphones (See Fig. 3 above). They are connected to three biosensors, on the first to third fingers of their non-dominant hand. Sensors capture heart rate and skin conductance; where variations in skin conductance are intended to indicate arousal, and heart rate indicates some combination of arousal and attentiveness . We rely on a Flexcomp encoder, skin conductance sensors, and a blood volume pulse (BVP) sensor, all from Though Technology Ltd.

We measure threat sensitivity by collecting the participants' physiological activity while being exposed to the photos. While sitting at the computer and watching the screen, participants are exposed to a 1-minute gray screen, followed by two sets of photos. The first two images are mundane objects to capture people's baseline responses and the second two are selected to measure threat sensitivity. The individual photos within the two blocks are randomized. These images will be relatively mild (e.g., a snake or barking dog) in accordance with the type of images one might see in a general circulation newspaper. Each photo is shown for 10 seconds, then there is a 10-second gray screen, and the process is repeated until they have seen the two randomized blocks of photos, a total of four photos. Note that this protocol is a standard way to measure threat sensitivity and it has been employed in a number of studies (e.g., Oxley et al. 2008).

Data were gathered using software built for work by **?**, and now used in ongoing work by Soroka, Patrick Fournier and Lilach Nir **?**. More details are available in the methodological appendix to that ongoing work, available here:
http://www.snsoroka.com/files/Method.Appendix.pdf.

Script for physiological procedures

All participants are guided through the physiological portion of the protocol using a relatively simple script. There are of course small variations based on questions from participants. The script we used is as follows:

Hello, I'm NAME. It is a pleasure to meet you. First, thank you very much for participating in our study. We really appreciate your help. This is where we will conduct this second part of the study. You will only be seated at this computer for a total of about four minutes.

Before we begin, I will briefly describe this portion of the study. The screen will display a series of four images. You simply need to watch them. The images are separated by a blank screen. The first blank screen will be longer than the screens you will see between the other photos. It simply needs to calibrate for a while before beginning. Once we start, the whole process will take about two minutes. When the screen says, "thank you," it is over; just let me know. [Note that if asked we describe the general goal of the study (understanding reactions to stimuli), but we do not talk about the hypothesis we are examining before the experiment (comparing reactions to neutral and threat stimuli).] If everything is clear and you don't have any questions, then we can proceed.

Please have a seat. Are you right-handed or left-handed? We will put the equipment on your non-dominant hand. First, this sensor will measure your heart rate. It goes on your middle finger. The velcro should be loose, but it should not be too tight, let me know if this is not comfortable. The next two sensors will measure your skin conductance. One goes on your index finger, the other goes on your ring finger. Are they too tight? We can check whether the sensors are working well by starting the software. The top line is heart rate. The bottom line is skin conductance. If you move too much, the sensors are affected, so please find a position where you can stay immobile during the next couple of minutes. Some people prefer to put their hand on the chair's armrest, some prefer to put it on their leg, some prefer to put it on the table. Chose the position you like best. Is the computer screen correctly inclined?
You can now put on the headphones. You can adjust their volume by clicking on these two buttons. The headphones are noise canceling, and help to avoid distractions. (After the photo experiment is over.) We can now take off the headphones and the sensors. Thank you once again for your participation. We are very grateful. Do you have any questions?

Threat photo manipulation check

In this section, we evaluate whether the threatening photos increased galvanic Skin Conductance Levels (SCL). Table 2 explores the relationship between SCL and photo content using an analysis in which each participant-photo combination is a case. There are four cases for each respondent, and the analysis relies on an OLS model with clustered standard errors accordingly. In the first model, we regress galvanic skin levels on binary variables for photos of the spoon, dog and snake, where the basket is the residual category. In the second, we add a variable that ranges from 1 to 4, and captures the order in which photos are viewed. Past work suggests that SCL tends to decrease over the course of an experiment, so this control may be important. Here, we find that it is statistically significant, and negative as expected. It also helps strengthen the estimated relationship between photos and SCL; so we focus here on interpreting the impact of the photos as estimated in the second model.
Doing so is straightforward: The spoon photo does not produce any significant change in SCL vis-a-vis the basket photo, but both the dog and snake photos do. Both these threat-themed photos produce an average increase of roughly .085 – to put that in context, the SCL measure ranges from roughly -.4 to + .45, with a standard deviation of .065. The average impact of the threat photos is thus both significant and substantive.

Table A: GSL, by photo contents and order

	Dependent variable:	
	Normalized Galvanic Skin Conductance	
	(1)	(2)
Spoon	0.013	0.007
	(0.010)	(0.009)
Dog	0.029***	0.090***
	(0.009)	(0.018)
Snake	0.020*	0.081***
	(0.008)	(0.016)
Order		−0.032***
		(0.006)
Constant	−0.008	0.075***
	(0.006)	(0.016)
Observations	516	516
R^2	0.027	0.087
Adjusted R^2	0.022	0.080
Residual Std. Error	0.064 (df = 512)	0.062 (df = 511)
F Statistic	4.820** (df = 3; 512)	12.192*** (df = 4; 511)
Note:		*p<0.05; **p<0.01; ***p<0.001

Table B: Right-left spending preferences and threat sensitivity

	Dependent variable:	
	Spending on Right - Left Issues	
	(1)	(2)
Threat Sensitivity	78.093	77.045
	(133.946)	(133.230)
Democrat		−27.802
		(15.803)
Republican		−16.759
		(20.390)
Constant	−93.231***	−73.647***
	(7.071)	(13.804)
Observations	97	97
R^2	0.004	0.036
Adjusted R^2	−0.007	0.005
Residual Std. Error	63.488 (df = 95)	63.112 (df = 93)
F Statistic	0.340 (df = 1; 95)	1.160 (df = 3; 93)
Note:		*p<0.05; **p<0.01; ***p<0.001

Table C: Crime-poor spending preferences and threat sensitivity

	Dependent variable:	
	spend_police.poor	
	(1)	(2)
Threat Sensitivity	73.214*	75.783*
	(36.035)	(35.423)
Democrat		−3.829
		(3.971)
Republican		6.550
		(5.001)
Constant	−8.681***	−7.706*
	(1.780)	(3.468)
Observations	117	117
R^2	0.035	0.084
Adjusted R^2	0.026	0.060
Residual Std. Error	17.563 (df = 115)	17.254 (df = 113)
F Statistic	4.128* (df = 1; 115)	3.475* (df = 3; 113)
Note:		*p<0.05; **p<0.01; ***p<0.001
